# Supplementary material for: The Relationship between Health Literacy and COVID-19 Vaccination Prevalence during a Rapidly Evolving Pandemic and Infodemic
Source: Vaccines (Basel). 2022 Nov 23;10(12):1989. doi: 10.3390/vaccines10121989 (PMC9785031; doi:10.3390/vaccines10121989)
Supplement: Supplementary file 1 [file vaccines-10-01989-s001.zip › vaccines-2049052-supplementary.pdf]

Table S1: Health Literacy Scales

Scale Name	Individual	Organizational	Functional	Interactive	Critical
Feeling understood and supported by healthcare providers (HPS)		X		X	
Having sufficient information to manage my health (HSI)	X		X		
Actively managing my health (AMH)	X			X	X
Social support for health (SS)	X			X	X
Appraisal of health information (CA)	X				X
Ability to actively engage with healthcare providers (AE)	X			X	
Navigating the healthcare system (NHS)		X		X	
Ability to find good health information (FHI)	X		X	X	
Understand health information well enough to know what to do (UHI)	X		X		

Table S2: Comparisons of Functional, Interactive, and Critical Health Literacy among County Areas

	Kruskall-Wallis H Test Statistic		
	Functional	Interactive	Critical
South Fulton – North Fulton	7.26*	8.91*	10.06*
South Fulton – Atlanta	10.49*	12.47*	13.34*
North Fulton – Atlanta	1.66	1.67	1.21

*Significant at $\alpha < .05$
